# Rapid self-assembly of DNA on a microfluidic chip

**DOI:** 10.1186/1477-3155-3-2

**Published:** 2005-02-18

**Authors:** Yao Zheng, Tim Footz, Dammika P Manage, Christopher James Backhouse

**Affiliations:** 1 Department of Electrical and Computer Engineering, 2^nd ^Floor, ECERF Building (9107 – 116St.) University of Alberta, Edmonton, Alberta, T6G 2V4 Canada; 2 Department of Medical Genetics, University of Alberta, Edmonton, Alberta, Canada

## Abstract

**Background:**

DNA self-assembly methods have played a major role in enabling methods for acquiring genetic information without having to resort to sequencing, a relatively slow and costly procedure. However, even self-assembly processes tend to be very slow when they rely upon diffusion on a large scale. Miniaturisation and integration therefore hold the promise of greatly increasing this speed of operation.

**Results:**

We have developed a rapid method for implementing the self-assembly of DNA within a microfluidic system by electrically extracting the DNA from an environment containing an uncharged denaturant. By controlling the parameters of the electrophoretic extraction and subsequent analysis of the DNA we are able to control when the hybridisation occurs as well as the degree of hybridisation. By avoiding off-chip processing or long thermal treatments we are able to perform this hybridisation rapidly and can perform hybridisation, sizing, heteroduplex analysis and single-stranded conformation analysis within a matter of minutes. The rapidity of this analysis allows the sampling of transient effects that may improve the sensitivity of mutation detection.

**Conclusions:**

We believe that this method will aid the integration of self-assembly methods upon microfluidic chips. The speed of this analysis also appears to provide information upon the dynamics of the self-assembly process.

## Background

There has been a rapid growth in the number of applications that are based upon DNA self-assembly, ranging from DNA microarrays (e.g. Affymetrix [1]) in the life sciences, through conformation-based mutation detection methods [2,3], to the ongoing development of DNA scaffolding methods of nanoassembly [4]. The control of the degree of DNA hybridisation requires elaborate and time consuming sample preparation (eg [5]) with methods that may require hours to achieve hybridisation [6], and on the order of an hour even within miniaturised systems [1,7]. However, a rapid method of controlling denaturation and renaturation within a microfluidic device would enable an inexpensive mutation detection method that could be performed within minutes.

Microfluidic devices or 'microchips' are photolithographically-defined networks of microchannels in glass where the microchannels are similar in size to conventional capillaries. These microchips provide compelling advantages in terms of speed, reagent usage and integration over conventional capillary or gel-based methods. The potential of the microfluidic chip has led to the use of terms such as "micro-total analysis systems" and "lab-on-a-chip". These microchips have been demonstrated in conjunction with a range of applications that integrate the polymerase chain reaction (PCR) and capillary electrophoresis (CE) methods with some reaching nanolitre or smaller scale volumes. A powerful advantage of the microchip approach is that it can implement much the same molecular biology protocols and reagents as used with conventional equipment, thereby allowing a wealth of established expertise to be transferred to the microscale.

Although the most effective method of mutation detection is sequencing, it is also by far the most expensive [8]. The microarray [8] technique, although powerful, is still handicapped by significant false positive rates and high cost [9]. Alternative methods based on DNA self-assembly are much faster than sequencing and these include single-strand conformation polymorphism (SSCP), denaturing high performance liquid chromatography (DHPLC) and heteroduplex analysis (HA). Although their cost has been shown to be far lower than sequencing, the achievable sensitivities (the percentage of mutations that are successfully detected) are only about 90 % [10,9].

Microfluidic chips may enable extremely high throughputs and high levels of integration. The achievement of this goal has been hindered by the lack of successful integrations of methods of mutation analysis based on single-stranded DNA (ssDNA) and double-stranded DNA (dsDNA) – likely due to the difficulties in controlling the degree of hybridisation on chip without time consuming thermal processing. A great advantage would be provided by a method of enabling microchip-based control of a rapid DNA self-assembly process.

The term wildtype is used to describe any given genetic sequence that does not contain mutations. Since individuals usually carry two copies of each gene, the genetic sequence of the two copies may be identical (homozygous) or may differ (heterozygous). DNA is normally double stranded, but under some conditions (e.g. high temperature), melts into single strands. Under other conditions, such as a lower temperature, these single strands will self-assemble into the double-stranded form again. The resulting double-stranded DNA is referred to as a homoduplex if the sequences are perfectly complementary, or a heteroduplex if the sequences are nearly complementary (i.e. a mutant sequence paired with a wildtype sequence). The misfit in a heteroduplex creates a "bulge" or "bubble" where the bases do not match and this affects the shape of the assembled molecule, typically lowering its velocity during electrophoretic movement, i.e. the heteroduplexes typically migrate more slowly than the homoduplexes. Any heterozygous sample will generate four different duplexes, two homoduplexes and two heteroduplexes, although the molecules often co-migrate so that fewer than 4 separate electropherogram peaks are resolved.

In the HA method, electrophoretic conditions are chosen in order to enhance the velocity differences between the duplexes so that the process of duplex self-assembly can be used to determine the presence of a heterozygous state (hence indicating the presence of a mutation). In SSCP, isolated strands of ssDNA find near-complementary sequences on the same strand, with the result that the strand folds upon itself in a sequence dependent manner forming new conformations. This is a simplistic description since ssDNA without self-similar sequences, and homoduplex dsDNA, may also take complex forms. Techniques such as HA that aim to separate homoduplex fragments from heteroduplex fragments often use some combination of thermally and chemically denaturing conditions to cause the partial melting of the duplex, resulting in a shift in mobility or chromatography column retention time that increases with the degree of mismatch.

Many medical diagnostics could be implemented on microchips if an effective implementation of a highly sensitive mutation analysis method could be integrated with PCR/CE. Considerable work has been done in developing SSCP [11] and HA [2,3,12]. An excellent review of such methods has been produced by Jin et al. [13]. The main drawback is the lower sensitivity of these methods. In macroscopic work Kozlowski and Krzyzosiak [5] and Kourkine *et al*. [14] greatly improved their sensitivities by combining SSCP and HA methods to develop capillary-based electrophoretic techniques with sensitivities of 90–94 % for SSCP and 75–81 % for HA. In a landmark analysis, Kourkine *et al*. achieved 100 % sensitivity by analysing denatured and non-denatured fragments in tandem. Despite being highly effective, the additional sample preparation required by these methods (i.e. separately preparing both single and double stranded DNA and maintaining this strandedness) complicates their implementation on microchips.

In this work, we present an electrophoretic method in which DNA is denatured in a microchip (with formamide) and, depending upon the sequence of applied voltages, can be prepared with a widely varying degree of hybridisation (i.e. from almost entirely ssDNA to almost entirely dsDNA). Given the small volumes involved within the microchip, diffusion time plays a small role and the reassembly process can be fast, with dsDNA obtained within minutes. The rapidity of the manipulation possible on this system allows some investigation of the dynamics of the reassembly, indicating that there are well-defined intermediate states where both ssDNA and dsDNA exist in the reassembly process.

We have applied our methods to the H63D and S65C mutations from the HFE gene associated with hereditary hemochromatosis (HH). The denaturation technique used enables a combined microchip-based method of HA and SSCP analysis.

## Results

### Heteroduplex Analysis

**Figure 1:**
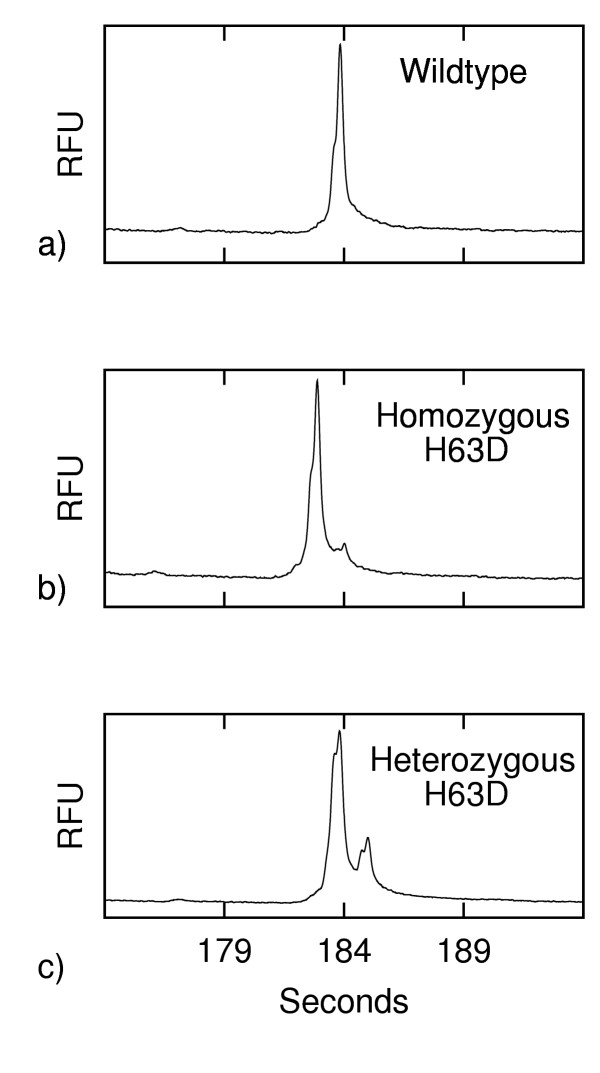
**Double-stranded DNA peak profiles prior to the addition of formamide (fluorescence in relative fluorescence units (RFU) vs. time). **a) wildtype, b) homozygous H63D mutant, c) heterozygous H63D mutant.

In our electrophoretic analyses with a double-T chip (described below), the dsDNA arrives at the detection point before the ssDNA (after about 105 s of separation, versus 190 s of separation for the ssDNA). As shown in Fig. 1, to demonstrate that our analysis conditions allow for the detection of mutations by means of HA, we analysed (undenatured) PCR products of a homozygous wildtype, a heterozygous and a homozygous H63D mutant. We found, as expected, that the heterozygous sample had two distinct peaks due to the transport of heteroduplexes as well as homoduplexes. However, the wildtype and homozygous mutant samples looked very similar, with the exception of a small peak following the main peak of the mutant sample. This small peak was only apparent with this sample and seems to indicate a PCR artefact. The size and shape of the bump remained consistent throughout the experiments and did not affect the peak intensities of either dsDNA or ssDNA. The bump is too small to add ambiguity when resolving the H63D mutation by HA and it should be noted that the emphasis of this work is on inducing the formation of dsDNA and ssDNA on-chip rather than upon improving mutation detection.

### Simultaneous Analysis of ssDNA and dsDNA

**Figure 2:**
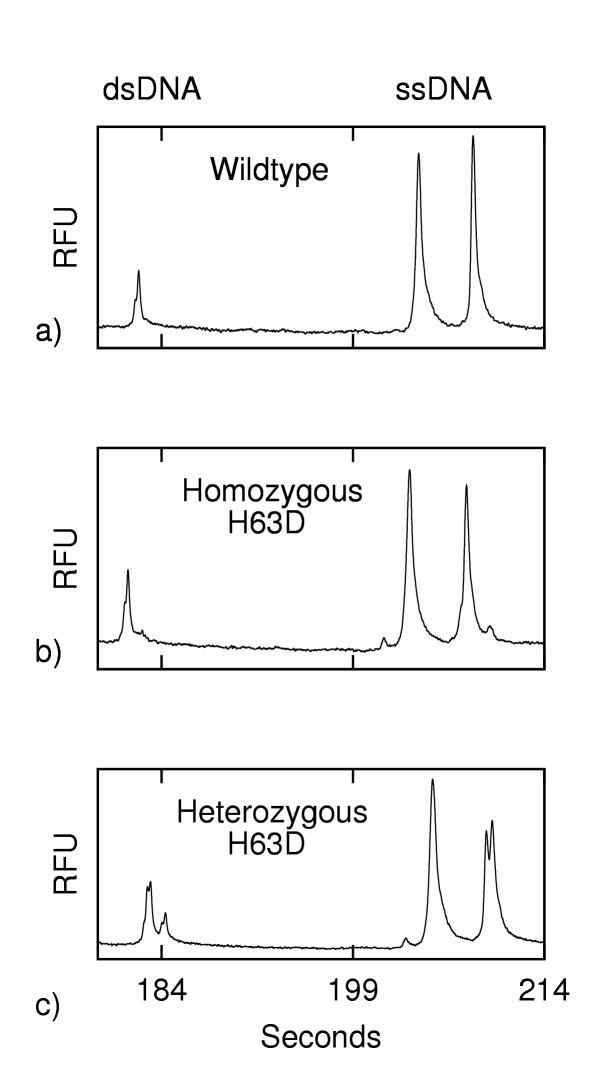
**Electropherograms of H63D (ss and ds DNA) following addition of formamide (fluorescence in relative fluorescence units (RFU) vs. time). **a) wildtype, b) homozygous H63D mutant, and c) heterozygous H63D mutant.

As expected, after the addition of formamide the electropherograms showed the presence of ssDNA peaks in addition to the dsDNA peaks (Fig. 2). The dsDNA profiles seen here are identical to those seen prior to the addition of formamide (Fig 1). The ssDNA peaks show differences in relative peak heights and in peak profile – and most notably the heterozygous sample shows a clefted peak. A comparison of the ssDNA profiles for the wildtype, homozygous mutant and heterozygous mutant would constitute a demonstration of SSCP analysis. Although the relative spacing of the ssDNA peaks differs between the wildtype and homozygous mutant, the most obvious difference is the clefted peak seen in the electropherogram of the heterozygous sample. This clefted peak was not present in the corresponding profiles of the homozygous samples (neither wildtype nor mutant). Under these conditions of electrophoresis, the mutational status of H63D is readily apparent. We have developed a combined HA and SSCP method and will report on it elsewhere (that report includes the detection of the common C282Y mutation). To our knowledge this is the first report of a method for performing combined on-chip HA and SSCP. Our emphasis here is on the ability to achieve rapid denaturation and renaturation processes on-chip.

### Reassembly of dsDNA

**Figure 3:**
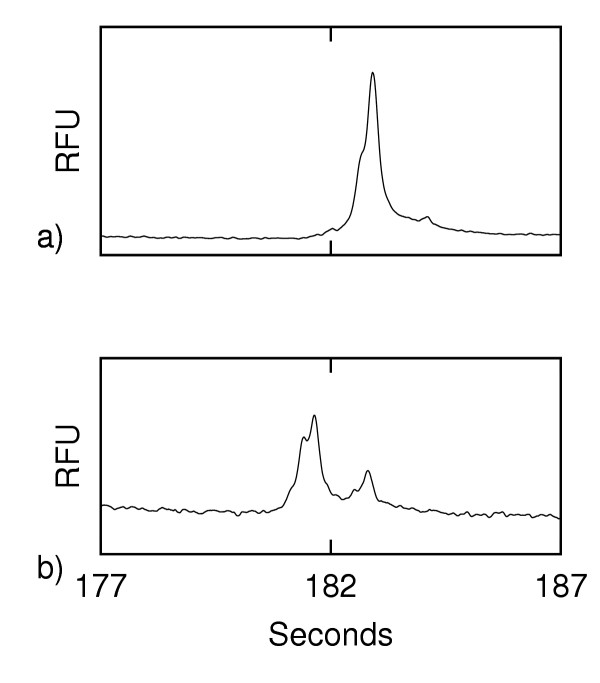
**Double-stranded DNA peak profile of the mixture of H63D homozygous mutant with wildtype prior to and after the addition of formamide (fluorescence in relative fluorescence units (RFU) vs. time). **a) prior to and b) after

In order to confirm that we are reassembling DNA on chip rather than denaturing to varying degrees we investigated the on-chip production of heteroduplexes from two samples of homoduplexes (i.e. homozygous) samples. Fig. 3a) shows the results of the analysis of a mix of the dsDNA from a homozygous H63D and its corresponding wildtype. This first analysis (done without the addition of formamide) showed a peak profile similar to that seen for the pure wildtype or homozygous mutant in Fig. 1 – i.e. no heteroduplexes are evident. We then added formamide to form a mixture in the sample well of homozygous mutant ssDNA with wildtype ssDNA. Once electrophoretically extracted from the sample well, the ssDNA reanneals to form heteroduplex mutants for analysis. As expected, the dsDNA profile of Fig. 3b) is that of the heteroduplex profile seen in Fig. 1 (the signal to noise ratio of this electropherogram is low because the sample is still primarily ssDNA). This indicates that the DNA is extracted from the formamide-rich sample well as ssDNA and reassembles to dsDNA in the microchip channels.

**Figure 4:**
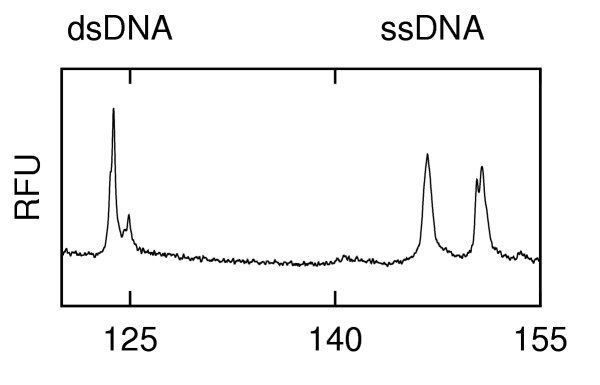
Separate injections of wildtype and homozygous H63D samples recombining on-chip (fluorescence in relative fluorescence units (RFU) vs. time)

The reassembly of ssDNA can also be shown by denaturating the wildtype and homozygous mutant in separate wells. The two denatured samples were injected simultaneously and their ssDNA mixed in the injection channel of a Y-chip (described below). Subsequent separation and detection showed peak profiles (Fig. 4) similar to those obtained with the heterozygous mutant for both HA and SSCP. This suggests that the method of denaturation used here is a powerful tool for comparing test samples, either in the same or in separate sample wells. The testing of the wildtype, homozgyous and heterozygous mutants could be conducted by injecting samples from the desired wells without reloading the chip. This would greatly improve throughput.

As will be described in the following section, by varying the electrophoretic parameters we can control the relative amount of dsDNA formed – a significantly larger amount could be obtained.

### Dynamics of DNA Reassembly

After demonstrating that reassembly occurred within the microchannels after extraction from the formamide-rich sample well, it was of interest to investigate how the sequence and timing of the sample extraction and analysis might affect the degree of rehybridisation. In the work presented thus far we used a 60 s injection (although 20 s would probably have sufficed) as a means of drawing sample directly from the sample well to the intersection, from whence it could be analysed.

**Figure 5:**
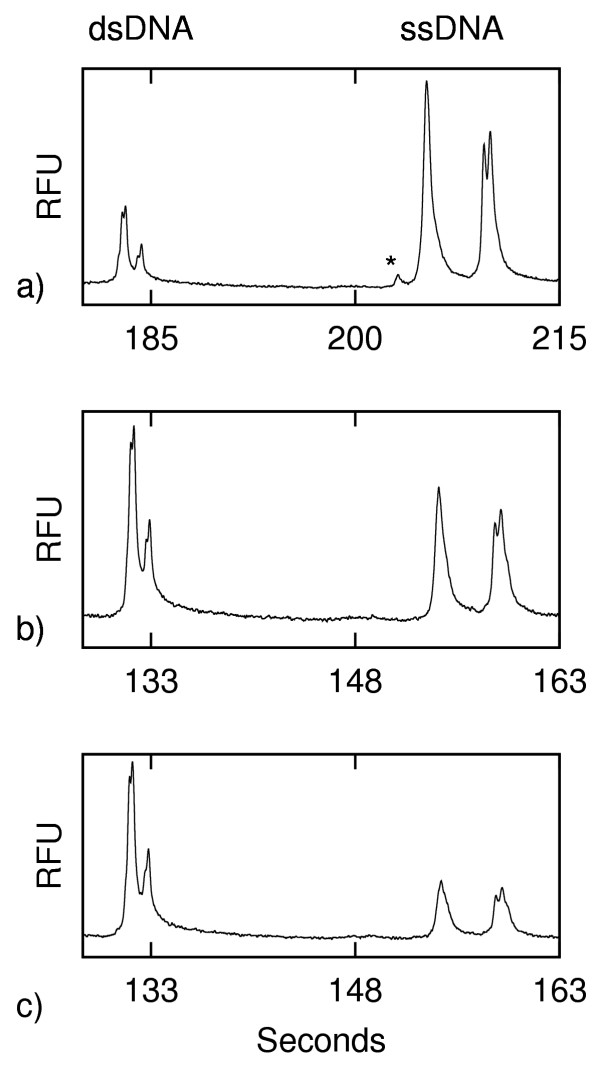
**Electropherograms after successive short injections of H63D heterozygous mutant DNA that show the change of ssDNA to dsDNA in the channels after leaving the formamide-rich environment of the sample well. (fluorescence in relative fluorescence units (RFU) vs. time). **a) H63D immediately after a 60 s injection. b) H63D after a subsequent 10 s injection c) H63D after a second subsequent 10 s injection

After the addition of formamide and an analysis of the resulting sample (60 s injection and 180 s separation), a series of analyses were performed wherein each short injection (10 s) with a lower electric field was followed by a 180 s separation. These short injections sampled DNA that had remained in the microchannel since its extraction during the 60 s injection from the first analysis. The time required for the DNA to travel from the sample well to the intersection with the applied electric field during the 10 s short injections was calculated to be approximately 53 s. (The short injections are carried out at a lower field than the initial injection.) Thus, the two short injections of 10 s each were not enough to bring in fresh samples from the sample well. Fig. 5 indicates that after the initial 60 s injection the dsDNA concentration steadily increases as rehybridization occurs in the microchannel. Depending on extraction timing (e.g. short injections vs. longer), the relative intensities of the ssDNA and the dsDNA can be varied by a factor of approximately 10, ranging from primarily ssDNA to primarily dsDNA. Further optimisation is possible with changes in microchip geometry. (Shorter injection channels would allow for more ssDNA to be introduced).

**Figure 6:**
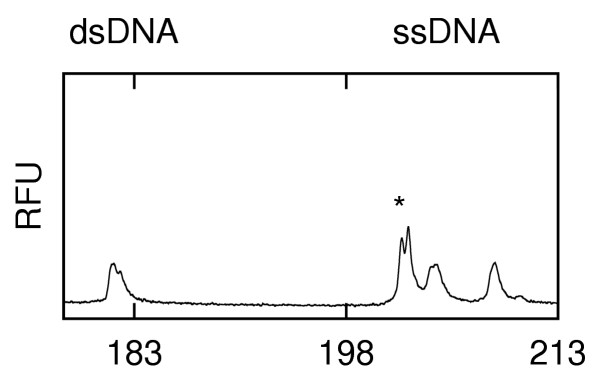
Electropherogram (ss and ds DNA) after initial 60 s injection of S65C heterozygous mutant DNA (fluorescence in relative fluorescence units (RFU) vs. time).

Another interesting feature of Fig. 5 is that following the addition of formamide, the first peak of the ssDNA (marked *) seen in the first analysis after a 60 s (Fig. 5a) injection is never present after a subsequent 10 s injection (Fig. 5b) although it can be recovered by another 60 s injection (not shown). The strength of this peak is strongly dependent upon the sample tested (as discussed below). This interesting phenomenon was observed with wildtype, homozygous and heteroduplex samples corresponding to H63D and S65C (data not shown) and the transient peak was clefted for heterozygous S65C (Fig. 6) and not clefted for H63D (Fig. 5a). It appears that this intermediate state may be used to investigate the dynamics of reassembly by a rapid microchip-based method.

## Discussion

The integration onto a microchip of an effective means of mutation detection is perhaps one of the most important technological barriers to the implementation of microchip-based medical diagnostics. The best means of attaining sufficiently high sensitivity is by integrating several existing methods of microchip-based mutation detection. The capillary-based analysis procedure developed by Kourkine *et al*. [14] is likely to be highly effective in conjunction with the microchip analysis of prepared samples, but since the procedure is based upon the thermal processing (95°C and snap cooling) of diluted PCR products, the integration of this processing onto the microchip may be problematic. The present method allows for such integrations, thereby enabling the mutation analysis throughputs predicted by Medintz *et al. *[15] – throughputs as much as 100 times higher that those presently attainable. Another issue is that of signal to noise ratios – rather than dilute our sample (possibly weakening its signal strength) we can analyse the sample essentially undiluted. Moreover, we can enhance the signal strength, as we choose, for either the ssDNA or the dsDNA.

As demonstrated here, this method also allows on-chip comparisons of one type of DNA with another. A common problem encountered with HA methods is that they cannot distinguish homozygous mutant from homozygous wildtype – the present technique would allow an on-chip comparison of these samples to produce heteroduplexes that will then indicate the mutational status.

The on chip denaturation is produced through the addition of formamide. The melting temperature for this sequence of DNA following the addition of formamide was found to be approximately room temperature, as determined by

T_m _= 81.5 + 16.6(log M) + 0.41 (% G + C) - 0.72 (% formamide)     (1)

where T_m _is the melting temperature in degrees Celsius, M is the monovalent salt molarity, (% G + C) is the percent of the guanine and cytosine in the DNA strand of interest, and (% formamide) is the percentage of formamide added [16]. The melting of DNA was confirmed by forming heteroduplexes on-chip.

The ability to quickly re-hybridise on chip allows for rapid investigation of self-assembly mechanisms. In addition, this re-hybridisation enables the formation of duplexes made from a sample and a set of DNA references – i.e. DNA self-assembly within a microchip could be used to form duplexes that, under electrophoretic analysis, would show the results of comparing the sample DNA with each type of DNA in the reference set. This could avoid the need for DNA sequencing.

The rapidity of our method appears to provide additional information upon short-lived conformations. Although we have added a thermal re-annealing step as part of our PCR protocol, that step does not affect the results of analysis after adding formamide – i.e. by re-annealing on-chip the thermal reannealing is not needed. The thermal re-annealing stage was added to allow the direct comparison of heterozygous samples from the PCR with heterozygous samples after on-chip reassembly. After adding formamide, the electropherogram of the first separation analysis following any long injection shows a clearly defined transient peak. For H63D samples the transient peak is a single peak, whereas for S65C the transient peak is clefted. We have found that the transient peaks vary in size significantly depending upon the electrophoretic and PCR protocols used. Initially we had assumed that this transient peak indicated that the reassembly of the DNA was not 'random' but instead hybridised first in a high-melting point region, and only slowly thereafter. In this model, the presence of the split-peak would provide information upon the location of the mutation. This suggests that mutation S65C is within the higher melting point domain, while the H63D is not. However, as determined by the Meltmap program (generously provided by L. Lerman (MIT)), neither the H63D nor S65C mutations were within the high melting point region of the exon (data not shown).

Several research groups have reported artefacts that arise from ssDNA-primer interactions [14,17-19]. Kourkine *et al. *[14,18] reported that primer-ssDNA complexes can give rise to extra peaks during SSCP. They performed tests with samples of PCR-amplified DNA with and without the removal of the PCR-primers after the amplification step and found that the presence of primers led to the appearance of extra peaks [18]. A reduction in primer concentration during PCR also proved to be effective in minimizing the appearance of these peaks. Kozlowski and Krzyzosiak [19] have reported similar effects and suggested that the primer-ssDNA complex may have a different mobility simply because of its changed mass, or perhaps due to a change in conformation induced by the binding. In the context of SSCP, they discussed two approaches for dealing with this effect 1) remove it through purification so as to obtain simpler profiles or 2) use the effect to advantage by achieving higher sensitivity in the detection of mutations. Hennessy *et al. *[17] performed similar tests and reported that variations in primer concentration are the likely source of irreproducible SSCP profiles. They too suggested that this effect could be used to increase the sensitivity of SSCP.

We therefore suggest that the transient peak is due to the pairing of one product strand with one primer as a result of the renaturation process. The primer-ssDNA complex is primarily ssDNA with a small region of dsDNA at the end(s) of the strand. It is therefore expected to migrate with similar mobility as the ssDNA peaks. The disappearance of the transient peaks with the subsequent short injections may be a result of the complementary single strand binding and displacing the primer. However, the presence of the transient peaks may still provide useful information. The differences in the transient peaks (cleft versus no cleft) between S65C and H63D suggest that their shape may be dependent on the position of the mutation and that the position greatly affects the transport of the transient form of DNA. Thus, the phenomenon of the transient may be a general behaviour that could provide additional mutational information. In corroboration of past work by others [17,19], it therefore appears that the primer effects do provide mutational information. Moreover, this effect can be produced or avoided depending on whether the desire is to avoid the more complex profiles or to use them to achieve higher sensitivity.

## Conclusion

We have developed a method of rapidly disassembling and re-assembling DNA within a microfluidic chip, allowing us control over the relative amount of ss and dsDNA and enabling the performance of rapid hybridisations under electrophoretic control. It has been reported that, when combined, HA and SSCP can provide sensitivities of 100% (e.g. [14]). In our work to date we have tested a large number of samples, predominantly of HFE, BRCA1 and BRCA2 sequences, and representing approximately several dozen different sequences. All samples containing a mutation have had their mutational status detected by at least one method. We expect then that the sensitivity of the combined methods will be close to 100%. We are now applying this method as part of a study of the application of DNA self-assembly based mutation detection methods (HA and SSCP) to the implementation of highly integrated microchips for performing medical diagnostics.

The present work is also an early step towards directing and studying DNA self-assembly within microfluidic systems. The method applied here could be improved significantly by shortening the injection and separation channels and ultimately may even assist in providing the control needed to direct the assembly of DNA-based nanosystems within microfluidic channels.

## Methods

### Samples

Volunteers who had given informed consent donated lymphocytes from which DNA was extracted and purified by using phenol-chloroform-isoamyl alcohol extractions [20] or the QIAmp DNA Blood kit (QIAGEN, Mississauga, ON). The purified DNA was solubilized in a Tris-EDTA buffer (TE, pH 8.0) and stored at 4°C. All genotypes were confirmed on an ABI Prism 377 Slab Gel Sequencer (Applied Biosystems, Streetsville, ON), using an ABI Prism BigDye Terminator v3.0 Ready Reaction Cycle Sequencing Kit with AmpliTaq DNA Polymerase (Applied Biosystems).

The two mutations tested were H63D and S65C, from HFE Exon 2. PCR was performed on 25 μL reactions of both mutations. Thermal cycling was performed on all the samples as follows: 94 C for 2 min, 35 cycles of (94°C for 30 s, 55°C for 30 s, 72°C for 30 s), and finally 72°C for 10 min, 4°C thereafter. For H63D and S65C, the PCRs are performed with 5 μL of 30 ng/μL of genomic template DNA, 2 μL of 5 μmol/L each of HEX-HFE-2F primer and H63DR primer (Table 1), 2 μL each of 10 mmol/L dNTPs, 0.75 μL of 50 mmol/L of MgCl_2_, 2.5 μL of 10× PCR reaction buffer and 0.5 μL of Platinum Taq DNA Polymerase. All samples were re-annealed following PCR by first heating at 95°C for 3 min, followed by a subsequent ramping down of temperature by 1°C per minute until 65°C. The samples were then stored at -20°C.

**Table 1:** Primers Used for PCR

Amplicon – Primer	Sequence	5' Label	Final Concentration
*HFE *Exon 2 – forward	5'-TCA GAG CAG GAC CTT GGT CTT TCC-3'	HEX	0.4 μM
*HFE *Exon 2 – reverse	5'-CAT ACC CTT GCT GTG GTT GTG ATT-3'	N/a	0.4 μM

### Reagents

PCR reagents (polymerases, buffers and primers) were obtained from Invitrogen (Burlington, ON). GeneScan™ polymer was used for microchip electrophoresis and obtained from PE Applied Biosystems (Foster City, CA). A polymer consisting of 5% GeneScan polymer and 10% glycerol (5GS10G), commonly used for SSCP, was made. Tris borate (Fisher Scientific, Fairland, NJ) with EDTA (Merck KGaA, Darmstadt, Germany) was used as the running buffer in concentrations of 1× and 0.1×. Glycerol (Sigma, Saint Louis, MO) is also added to each in 10% and 1% concentrations respectively (1 × TBE10G and 0.1 × TBE1G). De-ionised formamide (minimum 99.5%) was obtained from Sigma (F9037, Saint Louis, MO). The formamide was aliquotted and kept frozen until required.

### Microchip Electrophoresis

**Figure 7:**
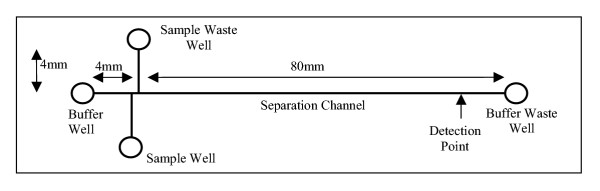
Glass microchip (Micralyne Inc.) with double-T intersection.

**Figure 8:**
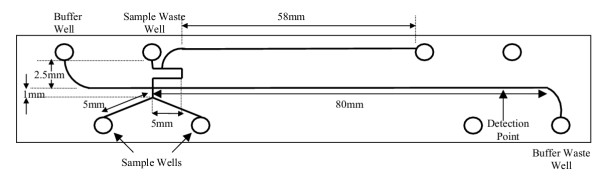
Glass microchip (Micralyne Inc.) with Y-shaped intersecting channels.

The microchips were purchased from Micralyne (Edmonton, AB) and unless otherwise mentioned were a 4 port double T design (Fig. 7) consisting of 4 reservoirs (or wells) linked by two microchannels. One microchannel served as a separation channel approximately 80 mm in length and was nominally 50 μm wide and 20 μm deep. In order to demonstrate control of on-chip mixing we also used an 8-port Y-chip with 8 reservoirs, 2 of which are not connected by any channel and with a third reservoir connected by a 58 mm channel that was unused in this work (Fig. 8). Electrophoresis upon microchips was performed using the Microfluidic Tool Kit (μTK, Micralyne) as described previously [2], with a laser induced fluorescence (LIF) system that provides excitation at a wavelength of 532 nm and detection at 578 nm. The LIF signal was recorded by the μTK with sampling at 200 Hz and these data were recorded to a PC running a compiled LabVIEW interface (supplied by Micralyne).

### Microchip Loading and Electrophoresis

The microchip was loaded with 5GS10G polymer without any pre-treatment. The sample well was loaded with 2.6 μL of 0.1 × TBE1G followed by 0.4 μL of DNA sample and mixed. The remaining wells were loaded with 3 μL of 1 × TBE10G. In the case of the Y-chip, 0.4 μL of wildtype DNA was added to 2.6 μL of 0.1 × TBE1G in the first sample well and mixed. The second sample well was filled with 2.6 μL of 0.1 × TBE1G and 0.4 μL of homozygous mutant. The operation of the μTK (injection and separation) was automated through the use of the LabVIEW interface. LIF detection took place 76 mm downstream from the intersection. We have found that the reproducibility of the peak arrival times is within 2 per cent from one run to the next. As such we have not needed to introduce size standards.

#### Injection

The sample DNA was brought from the sample well to the intersection and onto the sample waste well by applying 500 V/cm for 60 s. No initial injection was done with the Y-chip prior to denaturation. During this process the buffer and buffer waste well are left electrically disconnected. In doing so the intersection of the two (three) channels is filled with the sample DNA. This stage is referred to as an injection due to the injection of DNA into the separation channel in the sharply defined volume of the intersection of the channels.

#### Separation

Immediately following injection, the DNA caught within the intersection is separated by applying 714 V/cm for 180 s between the buffer and buffer waste wells. During this step, the sample and sample waste wells are left electrically disconnected. The effective separation distance was 76 mm from the intersection.

#### Denaturation

After the initial run on the 4-port chip, 1.5 μL of the sample mixture was removed and 1.5 μL of formamide was added and mixed. Following Howley *et al. *[16], this is sufficient to denature the DNA with a melting temperature of approximately 25.7°C. Since Fig. 4 clearly shows formation of heteroduplexes, we take this to indicate that the temperature was high enough to allow strands to interchange. Another run was then done with the same parameters as above. In the case of the Y-chip, denaturation of each sample was done immediately following the addition of the samples to the wells. A voltage of 400 V was applied between the sample and sample waste wells during a 60 s injection followed by a separation of 180 s. Subsequent electrophoretic runs followed with 10 s injections at 125 V/cm and 180 s of separation at 714 V/cm for both the 4-port and Y-chip. No additional mixing of the two samples for the Y-chip were required

## Authors' Contributions

YZ performed the experimental work with some assistance from TF. DM performed additional protocol development. CB provided overall direction. All authors contributed to the writing of the manuscript and all made substantial contributions to the work.
